# Expression, purification and crystal structure determination of a ferredoxin reductase from the actinobacterium *Thermobifida fusca*

**DOI:** 10.1107/S2053230X2000922X

**Published:** 2020-07-28

**Authors:** Jhon Alexander Rodriguez Buitrago, Thomas Klünemann, Wulf Blankenfeldt, Anett Schallmey

**Affiliations:** a Institute for Biochemistry, Biotechnology and Bioinformatics, Technical University Braunschweig, Spielmannstrasse 7, 38106 Braunschweig, Germany; b Structure and Function of Proteins, Helmholtz Centre for Infection Research, Inhoffenstrasse 7, 38124 Braunschweig, Germany

**Keywords:** ferredoxin reductase, *Thermobifida fusca*, cytochrome P450

## Abstract

The ferredoxin reductase FdR9 from *Thermobifida fusca*, a member of the oxygenase-coupled NADH-dependent ferredoxin reductase (FNR) family, catalyses electron transfer from NADH to its physiological electron acceptor ferredoxin. It forms part of a putative three-component cytochrome P450 monooxygenase system in *T. fusca* comprising CYP222A1 and the [3Fe–4S]-cluster ferredoxin Fdx8 as well as FdR9. Here, FdR9 was overexpressed and purified and its crystal structure was determined at 1.9 Å resolution. The overall structure of FdR9 is similar to those of other members of the FNR family and is composed of an FAD-binding domain, an NAD-binding domain and a C-terminal domain. Activity measurements with FdR9 confirmed a strong preference for NADH as the cofactor. Comparison of the FAD- and NAD-binding domains of FdR9 with those of other ferredoxin reductases revealed the presence of conserved sequence motifs in the FAD-binding domain as well as several highly conserved residues involved in FAD and NAD cofactor binding. Moreover, the NAD-binding site of FdR9 contains a modified Rossmann-fold motif, G*x*S*xx*S, instead of the classical G*x*G*xx*G motif.

## Abstract 2

The structure of the FAD-containing ferredoxin reductase FdR9 from *Thermobifida fusca* was obtained at 1.9 Å resolution.

## 1. Introduction   

Ferredoxin reductases (FdRs) are essential components of the electron-transfer chains of three-component cytochrome P450 monooxygenases (Hannemann *et al.*, 2007 ▸). FdR9 from the actinobacterium *Thermobifida fusca* belongs to the oxygenase-coupled NADH-dependent ferredoxin reductase family of FAD-dependent electron-transfer enzymes [ferredoxin-NAD(P)H reductases; EC 1.18.1.3] (Vorphal *et al.*, 2017 ▸). It mediates the transfer of two electrons from NADH to an iron–sulfur cluster-containing ferredoxin via two successive one-electron transfer steps (Medina & Gómez-Moreno, 2004 ▸). Electron transfer between the ferredoxin reductase and the ferredoxin requires the formation of a ternary NADH–FdR–Fdx complex (Deng *et al.*, 1999 ▸; Kuznetsov *et al.*, 2005 ▸).

*T. fusca* is a moderately thermophilic soil bacterium (Bachmann & McCarthy, 1991 ▸) and is a rich source of thermostable enzymes for application in biocatalysis (Wilson, 2004 ▸). In the genome of *T. fusca* (Lykidis *et al.*, 2007 ▸), the gene coding for FdR9 (*Tfu_1273*) lies adjacent to the genes encoding CYP222A1 (*Tfu_1274*) and a putative [3Fe–4S]-cluster ferredoxin named Fdx8 (*Tfu_1275*). Based on this genomic placement, FdR9, Fdx8 and CYP222A1 are believed to form a three-component cytochrome P450 monooxygenase system. Here, we report the heterologous production, purification, crystallization and structure determination of FdR9, one of the physiological protein partners of this three-component system. The aim of this study was to provide a structural basis for further investigations of the intermolecular electron-transfer processes between FdR9 and Fdx8 as well as the associated protein–protein interactions.

## 2. Materials and methods   

### 2.1. Macromolecule production   

The gene encoding FdR9 (*Tfu_1273*) was provided by Professor Vlada Urlacher (Institute of Biochemistry II, Heinrich-Heine University, Düsseldorf, Germany) in the plasmid pET-22b(+). The gene was subcloned from pET-22b(+) into the vector pET-28a(+) using the restriction enzymes NdeI and EcoRI to include an N-terminal His_6_ tag fused to the resulting protein and a thrombin recognition site between the His tag and the FdR9 sequence. FdR9 was overexpressed in *Escherichia coli* C43(DE3) cells (Miroux & Walker, 1996 ▸; Dumon-Seignovert *et al.*, 2004 ▸) transformed with the *Tfu_1273*-containing pET-28a(+) vector. For functional expression, the bacteria were grown in 1 l TB medium supplemented with 50 mg l^−1^ kanamycin at 37°C. At an OD_600_ of 0.8, expression was induced by the addition of isopropyl β-d-1-thiogalactopyranoside to a final concentration of 0.5 m*M*. After expression for 16 h at 240 rev min^−1^ and 37°C, the culture was harvested by centrifugation (15 min at 4400*g* and 4°C). The cell pellet was resuspended in 24 ml lysis buffer (20 m*M* Tris pH 8.0, 300 m*M* NaCl) in the presence of protease inhibitors (one tablet of Complete EDTA-free per 50 ml; Sigma–Aldrich). After sonication (nine cycles of 20 s at an amplitude of 60% followed by a 10 s pause on ice using a Vibra-Cell VCX130, Sonics & Materials, USA) for cell disruption and subsequent centrifugation (15 min, 10 000*g*, 4°C) to remove cell debris, FdR9 was found in the soluble fraction. The His_6_-tag-containing FdR9 was purified by affinity chromatography on an ÄKTAprime FPLC system (GE Healthcare, Freiburg, Germany) using a 5 ml HisTrap column (GE Healthcare). The bound protein was eluted using a linear imidazole gradient (0–0.5 *M*) in five column volumes (CV). Selected FdR9-containing fractions were combined for incubation with the Thrombin CleanCleave Kit (Sigma–Aldrich) and were then dialyzed against 20 m*M* Tris pH 8.0, 40 m*M* NaCl at 4°C for 16 h. Dialyzed samples were again loaded onto a HisTrap column (GE Healthcare) to remove the cleaved His tag. The FdR9-containing flowthrough was loaded onto a 5 ml HiTrap Q HP column (GE Healthcare) and elution was performed using a linear NaCl gradient (0.04–1 *M*) in 5 CV. Selected FdR9-containing fractions were concentrated by ultrafiltration using a 30 kDa cutoff membrane (Amicon Ultra-15, Merck) and were further purified by gel filtration on a Superdex 75 26/60 column (GE Healthcare) using 20 m*M* Tris pH 8.0, 300 m*M* NaCl, 1 m*M* dithiothreitol. Purified FdR9 was obtained with a yield of 60 mg per litre of culture and displayed a yellow color indicative of the flavin cofactor. A high degree of purity was confirmed for FdR9 by the observation of a single band on a 12% SDS–PAGE gel. Macromolecule-production information is summarized in Table 1 ▸.

### 2.2. Crystallization   

**Figure 1:**
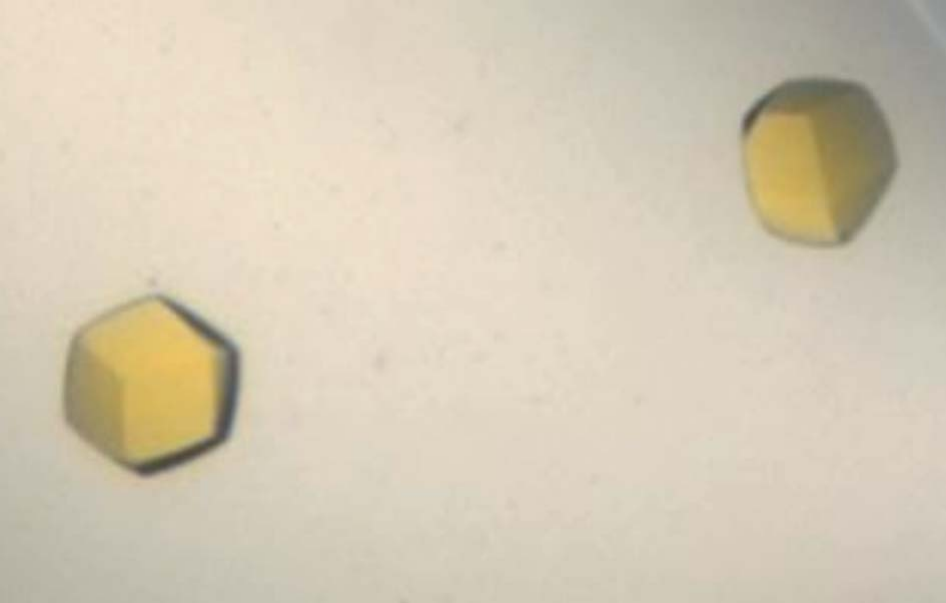
Crystals of ferredoxin reductase FdR9 from *T. fusca*, with dimensions of 50 × 50 × 50 µm, obtained from Index screen condition C6.

Initial crystallization trials were carried out at room temperature in 96-well Intelli-Plates (Art Robbins Instruments, Sunnyvale, California, USA) with the Index screen (D’Arcy *et al.*, 2003 ▸) using the sitting-drop vapor-diffusion method. The droplet was initially formed of 200 nl of a solution consisting of 42 mg ml^−1^ FdR9 in 20 m*M* Tris pH 8.0, 300 m*M* NaCl, was mixed with 200 nl reservoir solution using a pipetting robot (Honeybee 963, Genomic Solutions, Huntingdon, UK) and was then equilibrated against 60 µl reservoir solution. Crystals of FdR9 were obtained in condition C6 of the Index screen, consisting of 1.5 *M* ammonium sulfate, 0.1 *M* NaCl, 0.1 *M* bis-Tris pH 6.5. The crystals were yellow-colored as is typical for native FNR crystals owing to the presence of the flavin cofactor (Morales *et al.*, 2000 ▸; Fig. 1 ▸). Trials to optimize the crystal quality by varying the precipitant concentration or the pH did not result in better diffracting crystals. Crystallization information is summarized in Table 2 ▸.

### 2.3. Data collection and processing   

FdR9 crystals were harvested using a nylon loop (Hampton Research) and were soaked in reservoir solution containing 20%(*v*/*v*) 2,3-(*R*,*R*)-butanediol prior to flash-cooling in liquid nitrogen. 3600 images were collected by the oscillation method with a range of 0.1° per image on a Dectris PILATUS 6M-F detector using single-wavelength synchrotron radiation on beamline X11 at PETRA III of the Deutsches Elektronen-Synchrotron (DESY), Hamburg, Germany. Reflection-image processing was performed using *DIALS* (Winter *et al.*, 2018 ▸) and *AIMLESS* (Evans & Murshudov, 2013 ▸) from the *CCP*4 suite (Winn *et al.*, 2011 ▸). Data-collection and processing statistics are summarized in Table 3 ▸.

### 2.4. Structure solution and refinement   

The initial phases were obtained by molecular replacement using *MrBUMP* (Keegan & Winn, 2008 ▸) executing *Phaser* (McCoy *et al.*, 2007 ▸) and using the atomic coordinates of putidaredoxin reductase (PDB entry 1q1w; Sevrioukova *et al.*, 2004 ▸) as a search model. Refinement was performed by alternating rounds of *REFMAC*5 (Murshudov *et al.*, 2011 ▸) and manual adjustments in *Coot* (Emsley *et al.*, 2010 ▸). The final refinement steps were performed with *phenix.refine* (Afonine *et al.*, 2012 ▸), including TLS refinement and the addition of riding H atoms. The FdR9 diffraction data and coordinates were deposited in the Protein Data Bank (PDB; Berman *et al.*, 2002 ▸) as PDB entry 6tuk. Representations of the structures were generated with *PyMOL* version 2.1.1 (Schrödinger, New York, USA). Refinement statistics are listed in Table 4 ▸.

### 2.5. Reductase activity assay   

The activity of FdR9 with NADH and NADPH as cofactors was determined spectrophotometrically by measuring the decrease in the ferricyanide concentration at 420 nm (∊_420_ = 1.02 m*M* ^−1^ cm^−1^; Roome *et al.*, 1983 ▸). Each 1 ml reaction contained 0.5 m*M* K_3_Fe(CN)_6_, an appropriate amount of FdR9 (3.7 µg for the reaction with NADH and 37 µg for the reaction with NADPH) and 0.5 m*M* NADH or NADPH in 50 m*M* potassium phosphate buffer pH 7.4. Measurements were performed at ambient temperature using a Cary 60 instrument (Agilent, Heilbronn, Germany).

## 3. Results and discussion   

### 3.1. Structural overview of FdR9   

**Figure 2:**
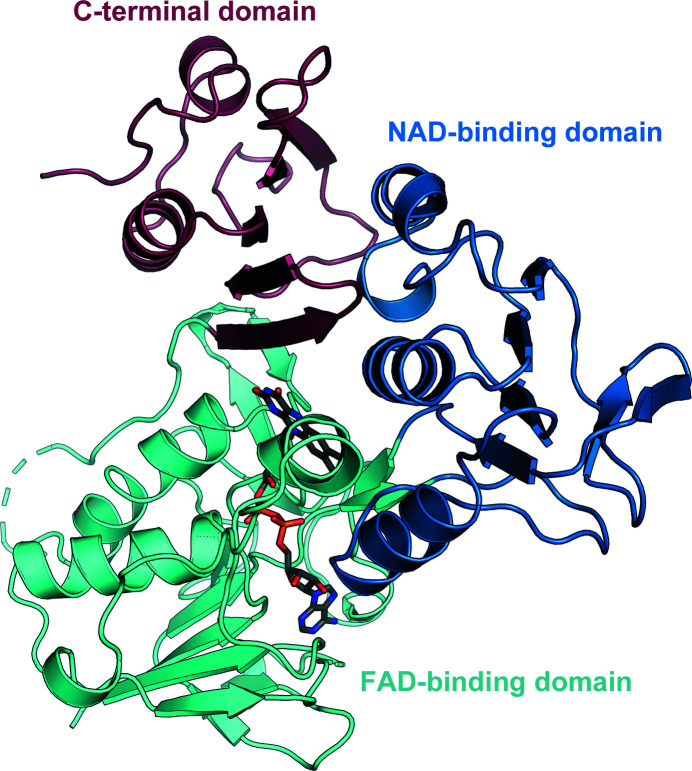
Overall structure of FdR9. The FAD-binding, NAD-binding and C-­terminal domains are colored cyan, light blue and light pink, respectively.

FdR9 crystallized in space group *P*4_1_32, with one monomer in the asymmetric unit. The initial phases were obtained by molecular replacement using the structure of putidaredoxin reductase (PDB entry 1q1w; 26% amino-acid sequence identity) as a search model. The resulting electron-density map allowed the identification of the FAD molecule bound to the reductase. The final model of FdR9, refined to an *R*_cryst_ of 18.5% and an *R*_free_ of 20.0%, displays very good geometry, with no residues located in disallowed regions of the Ramachandran plot. The structure of FdR9 contains nine α-helices and 25 β-strands, which form three distinct domains (Fig. 2 ▸): an FAD-binding domain (residues 1–106 and 223–308), an NAD-binding domain (residues 107–222) and a C-terminal domain (residues 309–393). Structural comparison of FdR9 with other proteins using the *DALI* server (Holm & Sander, 1995 ▸) indicated that it is very similar to ferredoxin reductases from *Rhodopseudomonas palustris* (PuR), *Novosphingobium aromaticivorans* (ArR), *Pseudomonas* sp. (BphA4) and *P. putida* (PdR) (Table 5 ▸), all of which share the three-domain architecture shown in Fig. 2 ▸. The root-mean-square deviations (r.m.s.d.s) for C^α^ atoms of these protein structures from the structure of FdR9 ranged between 1.9 and 2.2 Å. PuR, ArR and PdR are part of three-component cytochrome P450 monooxygenase systems, like FdR9, but transfer electrons to [2Fe–2S]-cluster ferredoxins, whereas the physiological interaction partner of FdR9 seems to be a [3Fe–4S]-cluster ferredoxin. In contrast, BphA4 forms part of the three-component biphenyl dioxygenase system present in *Pseudomonas* sp. (Table 5 ▸).

### 3.2. FAD-binding domain   

**Figure 3:**
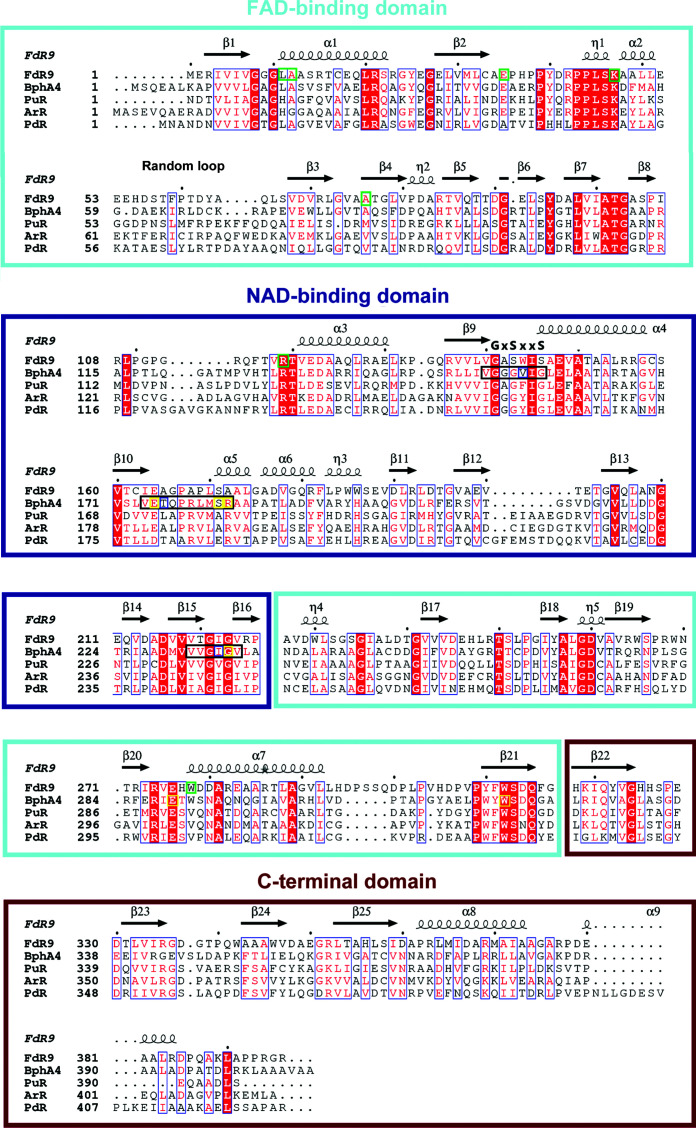
Multiple sequence alignment of FdR9 and homologous ferredoxin reductases obtained from a *DALI* search using the FdR9 structure as a template. Helices are represented by coils and β-sheets are shown as arrows. Columns with residues that are more than 70% similar according to physicochemical properties (threshold set to 0.7) are framed in blue and amino-acid residues with 100% identity are highlighted by a red background. FdR9 residues involved in hydrogen-bond interactions with the FAD molecule are framed in green boxes. The three loops of BphA4 interacting with the NAD molecule are framed in black boxes, and the residues of BphA4 interacting with the NAD molecule by hydrogen bonding and hydrophobic interactions are highlighted by yellow boxes. The PAM250 matrix was used for sequence alignment. The figure was rendered by *ESPript* 3.0 (Robert & Gouet, 2014 ▸).

**Figure 4:**
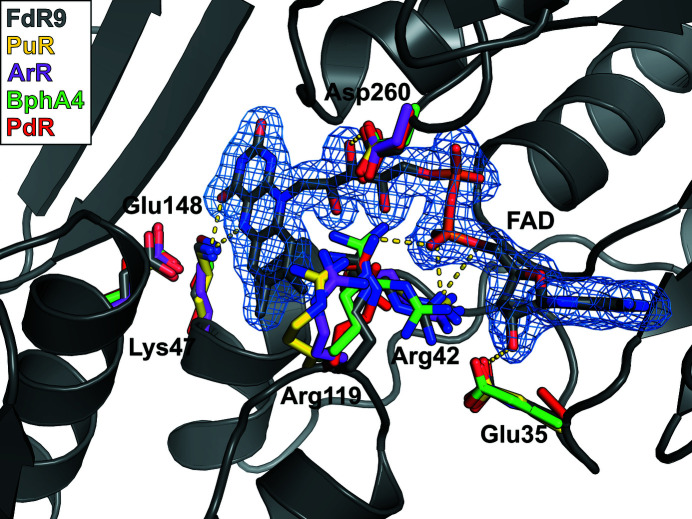
Superposition of the FAD-binding site of FdR9 (PDB entry 6tuk, gray) with the structures of other ferredoxin reductases [PDB entries 3fg2 (PuR), 3lxd (ArR), 2gqw (BphA4) and 1q1w (PdR)]. Residues forming hydrogen-bond interactions with the FAD cofactor are shown as sticks. Hydrogen bonds are indicated by dotted lines.

The FAD-binding domain of FdR9 adopts a typical α/β-fold consisting of two antiparallel β-sheets and one parallel β-sheet surrounded by four α-helices, as shown in Fig. 2 ▸. Sequence-alignment analysis of the FAD-binding domain reveals the presence of three highly conserved motifs: PY*x*RPPLSK, TS*x*P*x*_3_A*x*G and R*x*E*x*_4_A (Fig. 3 ▸). These motifs contain most of the amino acids that interact with the FAD cofactor. In FdR9, the FAD cofactor interacts with the protein through a network of hydrogen bonds to the FAD-binding domain involving amino-acid residues Leu11, Ala12, Glu35, Arg42, Lys47, Ala76, Arg119, Asp260 and Trp278 (Fig. 4 ▸), several of which are conserved among the ferredoxin reductases PuR, ArR, PdR and BphA4 (Fig. 3 ▸). The pyrophosphate moiety of FAD is stabilized by hydrogen bonds between the main-chain amide groups of Ala12 and Leu11 and O_1_P and between the side-chain NH_2_ group of Arg119 and O_2_A, as well as between the Asp260 side chain and the O_2_P and O_3′_ atoms of the riboflavin part. Moreover, Arg42 plays an important role in stabilizing the FAD cofactor through the formation of electrostatic interactions and hydrogen bonds with the O_1_A atom of the pyrophosphate moiety and the O_3_B atom of the adenosine ribose (Fig. 4 ▸). In PdR, this arginine is replaced by Leu45, while PuR, ArR and BphA4 also carry an arginine at the respective position (Figs. 3 ▸ and 4 ▸). Ala76 of FdR9 is involved in stabilization of the adenine through hydrogen bonding of the backbone carbonyl O atom to the N_6_A atom. The O^∊^ atom of Glu35 forms hydrogen bridges with the O_2_B and O_3_B atoms of the adenosine ribose. This glutamate is also conserved in PuR, ArR and BphA4, whereas an alanine is present at the corresponding position in PdR (Figs. 3 ▸ and 4 ▸). Trp278 stabilizes the isoalloxazine ring of the FAD cofactor by hydrogen bonding between its backbone NH and the O_2_ atom of the isoalloxazine ring. Lys47 in FdR9 forms a salt bridge with Glu148 and hydrogen bonds to the carbonyl O atom of Pro43 as well as to the O_4_ and N_5_ atoms of the isoalloxazine ring.

**Figure 5:**
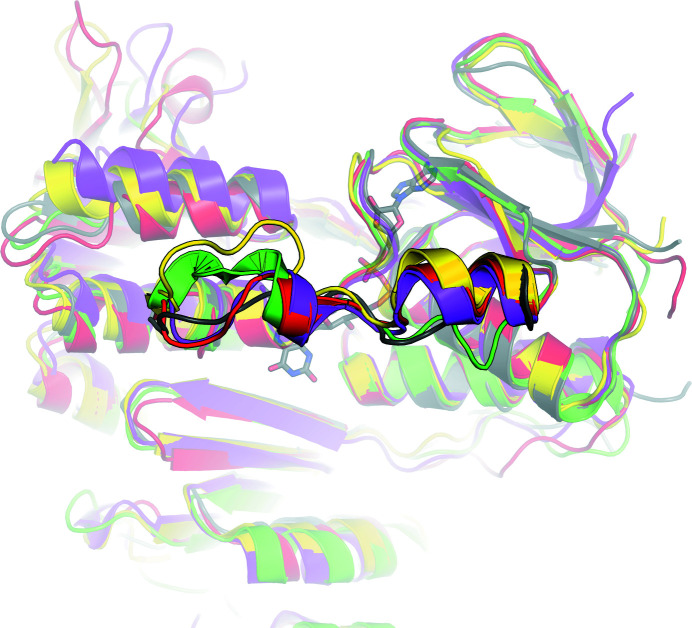
Superposition of the structural model of FdR9 (PDB entry 6tuk, gray) with the structures of other ferredoxin reductases [PDB entries 3fg2 (PuR) in yellow, 3lxd (ArR) in magenta, 2gqw (BphA4) in green and 1q1w (PdR) in red] to highlight differences in secondary structure in the random-loop region (residues 51–63 in FdR9).

Despite the fact that the overall fold of the FAD domain in FdR9 is highly similar to the structures of PuR, ArR, PdR and BphA4, with several conserved amino acids interacting with the FAD molecule, differences can also be observed. The first difference involves the presence or absence of secondary structure in the loop region (residues 51–63 in FdR9) of the five ferredoxin reductases. The short and longer helices found in this region in PdR, PuR, ArR and BphA4 correspond to a purely random loop in FdR9 (Fig. 5 ▸). It has previously been proposed that the absence of helix secondary structure in this region could influence the binding of FAD (Xu *et al.*, 2009 ▸). Another difference is the insertion of five amino acids (residues 295–300) in a surface loop in FdR9, while this loop is shorter in the other four reductases (Fig. 3 ▸). The electron density for residues 298–300 in this extended loop in FdR9 is not well resolved, indicating high flexibility.

### 3.3. NAD-binding domain   

**Figure 6:**
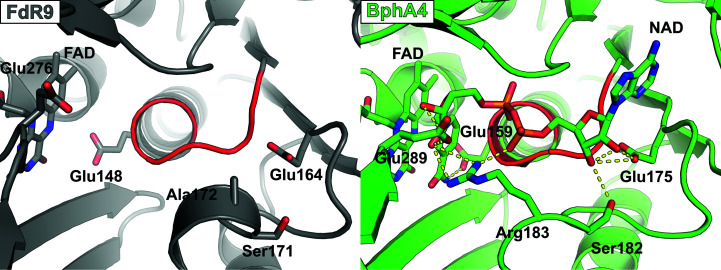
Structural comparison of the NAD-binding sites of FdR9 (PDB entry 6tuk) and the reductase BphA4 (PDB entry 1f3p) in complex with NAD^+^. The amino-acid residues of BphA4 interacting with NAD^+^ and the corresponding residues in FdR9 are labeled. The loops containing the G*x*G*xx*G motif of BphA4 and the G*x*S*xx*S motif of FdR9 are shown in red.

In our FdR9 crystallization experiments we did not attempt to co-crystallize FdR9 with the nicotinamide cofactor, and hence the structure of FdR9 presented here does not contain NAD. Nevertheless, sequence and structural analyses of the NAD-binding domain of FdR9 indicate the presence of a canonical Rossmann fold similar to previously reported complexes, in which the NAD molecule was shown to interact with three loops [corresponding to residues 140–146 (first loop), 164–172 (second loop) and 219–224 (third loop) in FdR9; Fig. 3 ▸] (Senda *et al.*, 2000 ▸). In PuR, ArR, PdR and BphA4, the first loop between β9 and α4 contains the typical G*x*G*xx*G motif indicative of nicotinamide cofactor binding, which is modified to **G**A**S**WI**S** in FdR9, with the last two glycines in the motif replaced by serines (Fig. 3 ▸). This change in the motif does not affect the folding of the respective loop, as can be seen in the structural comparison between FdR9 and BphA4 (Fig. 6 ▸). Previous studies proposed that the sequence motif G*x*G*xx*G was indicative of NAD specificity, whereas the motif G*x*G*xx*A is found in NADP-binding enzymes, although exceptions are known (Carugo & Argos, 1997 ▸; Hanukoglu, 2017 ▸), as would be the case for FdR9. In our studies, FdR9 was found to display a clear preference for NADH as the cofactor. Based on a ferricyanide reduction assay (Roome *et al.*, 1983 ▸), the specific activity of FdR9 with NADH was determined to be 104 U mg^−1^, whereas the specific activity with NADPH was only 1.2 U mg^−1^. Hence, the nicotinamide cofactor-binding site of FdR9 was compared in more detail with that of BphA4 (PDB entry 1f3p), which was crystallized with NAD bound in the active site. The comparison shows that FdR9 shares a similar fold at the entrance to the NAD-binding channel and an accessible nicotinamide-binding site above the isoalloxazine ring of the flavin cofactor. In BphA4, NAD interacts with Val155, Ile156 and Glu159 in the first loop (the corresponding residues in FdR9 are Trp144, Ile145 and Glu148) and with Glu175, Thr176, Ser182 and Arg183 in the second loop (corresponding to Glu164, Ala165, Ser171 and Ala172 in FdR9). Glu175 of BphA4 forms hydrogen bonds to the O_2_B and O_3_B atoms of the adenosine ribose as well as to Thr176. The negative charge of Glu175, which is conserved not only in FdR9 (Fig. 6 ▸) but also in PuR, ArR and PdR, has been proposed to enhance NAD cofactor specificity by repulsion of the phosphate of NADP (Hanukoglu, 2017 ▸). Ser182 in BphA4, which corresponds to Ser171 in FdR9, also forms a hydrogen bond to the adenosine ribose O_3_B atom. Residues in the third loop interacting with the NAD molecule (Ile235 and Gly236 in BphA4), as well as Glu289 and Trp320 of BphA4, are significantly conserved among the five ferredoxin reductases compared in Fig. 3 ▸. Interestingly, FdR9 has a threonine at position 220 (third loop), while PuR, ArR, PdR and BphA4 possess a hydrophobic residue (either valine or alanine) in the corresponding position. Of the 11 amino acids directly interacting with the NAD molecule in BphA4 by hydrogen bonding, eight are conserved in FdR9 (Fig. 3 ▸), whereas the proteins share an overall amino-acid sequence identity of only 30%. In contrast, other loop residues (especially in the first and second loop), which do not form direct hydrogen-bond interactions with the nicotinamide cofactor, display a lower degree of conservation.

Apart from this, FdR9 exhibits two significantly shorter surface loops in the NAD-binding domain compared with the other four ferredoxin reductases. This involves the loops between β8 and α3 as well as between β12 and β13 (Fig. 3 ▸). The latter in particular is seven, seven and nine amino acids longer in PuR, ArR and PdR, respectively. In PdR this loop is involved in crystallographic dimer formation, while PuR and ArR crystallize as monomers like FdR9 (Sevrioukova *et al.*, 2004 ▸; Xu *et al.*, 2009 ▸; Yang *et al.*, 2010 ▸). It is unclear, however, whether these differences in surface-loop lengths also have a possible physiological impact.

## Supplementary Material

PDB reference: Fdr9, 6tuk

## Figures and Tables

**Table 1:** Macromolecule-production information

Source organism	*T. fusca* strain XY
Expression vector	pET-28a(+)
Expression host	*E. coli* strain C43(DE3)
Complete amino-acid sequence of the construct produced	MERIVIVGGGLAASRTCEQLRSRGYEGELVMLCAEPHPPYDRPPLSKAALLEEEHDSTFPTDYAQLSVDVRLGVAATGLVPDARTVQTTDGELSYDALVIATGASPIRLPGPGRQFTVRTVEDAAQLRAELKPGQRVVLVGASWISAEVATAALRRGCSVTCIEAGPAPLSAALGADVGQRFLPWWSEVDLRLDTGVAEVTETGVQLANGEQVDADVVVTGIGVRPAVDWLSGSGIALDTGVVVDEHLRTSLPGIYALGDVAVRWSPRWNTRIRVEHWDDAREAARTLAGVLLHDPSSQDPLPVHDPVPYFWSDQFGHKIQYVGHHSPEDTLVIRGDGTPQWAAAWVDAEGRLTAHLSIDAPRLMIDARMAIAAGARPDEAALRDPQAKLAPPRGR

**Table 2:** Crystallization

Method	Sitting-drop vapor diffusion
Plate type	96-well Intelli-Plates
Temperature (K)	298
Protein concentration (mg ml^−1^)	42
Buffer composition of protein solution	20 m*M* Tris pH 8, 300 m*M* NaCl
Composition of reservoir solution	0.1 *M* NaCl, 0.1 *M* bis-Tris pH 6.5, 1.5 *M* ammonium sulfate
Volume and ratio of drop	200 nl, 1:1
Volume of reservoir (µl)	60

**Table 3:** Data collection and processing Values in parentheses are for the outer shell.

Wavelength (Å)	1.0332
Temperature (K)	100
Space group	*P*4_1_32
*a*, *b*, *c* (Å)	142.79, 142.79, 142.79
α, β, γ (°)	90, 90, 90
Resolution range (Å)	142.79–1.90 (1.94–1.90)
Total No. of reflections	2946416 (144375)
No. of unique reflections	39768 (2527)
Completeness (%)	100 (100)
Multiplicity	74.1 (57.1)
〈*I*/σ(*I*)〉	25.0 (2.0)
CC_1/2_	1.0 (0.704)
*R*_r.i.m._	0.018 (0.363)
Overall *B* factor from Wilson plot (Å^2^)	31.74

**Table 4:** Structure solution and refinement Values in parentheses are for the outer shell.

Resolution range (Å)	47.6–1.90 (1.97–1.90)
Completeness (%)	99.81 (100)
No. of reflections, working set	39634 (3887)
No. of reflections, test set	1951 (208)
Final *R*_cryst_	0.185 (0.243)
Final *R*_free_	0.200 (0.270)
No. of non-H atoms	
Protein	2949
Ligand	91
Solvent	233
Total	3273
R.m.s. deviations	
Bond lengths (Å)	0.010
Angles (°)	0.87
Average *B* factors (Å^2^)	
Protein	44.15
Ligand	44.48
Water	44.99
Ramachandran plot	
Most favored (%)	97.19
Allowed (%)	2.81
Rotamer outliers (%)	0.33

**Table 5:** *DALI* search against the PDB using the FdR9 structure

PDB code	Organism	Terminal oxygenase	Sequence identity (%)	Alignment length	*DALI Z*-score	Reference
3fg2 (PuR)	*Rhodopseudomonas palustris* strain CGA009	CYP199A2	27	380	46.8	Xu *et al.* (2009 ▸)
3lxd (ArR)	*Novosphingobium aromaticivorans* DSM 12444	CYP101D1, CYP101B1, CYP101C1, CYP101D2	30	383	47.5	Yang *et al.* (2010 ▸)
2gqw (BphA4)	*Pseudomonas* sp.	Biphenyl dioxygenase BphA1A2	30	382	47.2	Senda *et al.* (2007 ▸)
1q1w (PdR)	*Pseudomonas putida*	P450cam	26	386	46.6	Sevrioukova *et al.* (2004 ▸)
